# Association of Human Mobility Restrictions and Race/Ethnicity–Based, Sex-Based, and Income-Based Factors With Inequities in Well-being During the COVID-19 Pandemic in the United States

**DOI:** 10.1001/jamanetworkopen.2021.7373

**Published:** 2021-04-07

**Authors:** Suman Chakrabarti, Leigh C. Hamlet, Jessica Kaminsky, S. V. Subramanian

**Affiliations:** 1 Department of Global Health, University of Washington Schools of Public Health and Medicine, Seattle; 2 Department of Civil and Environmental Engineering, University of Washington College of Engineering, Seattle; 3 Harvard Center for Population and Development Studies, Department of Social and Behavioral Sciences, Harvard T.H. Chan School of Public Health, Boston, Massachusetts

## Abstract

**Importance:**

An accurate understanding of the distributional implications of public health policies is critical for ensuring equitable responses to the COVID-19 pandemic and future public health threats.

**Objective:**

To identify and quantify the association of race/ethnicity–based, sex-based, and income-based inequities of state-specific lockdowns with 6 well-being dimensions in the United States.

**Design, Setting, and Participants:**

This pooled, repeated cross-sectional study used data from 14 187 762 households who participated in phase 1 of the population-representative US 2020 Household Pulse Survey (HPS). Households were invited to participate by email, text message, and/or telephone as many as 3 times. Data were collected via an online questionnaire from April 23 to July 21, 2020, and participants lived in all 50 US states and the District of Columbia.

**Exposures:**

Indicators of race/ethnicity, sex, and income and their intersections.

**Main Outcomes and Measures:**

Unemployment; food insufficiency; mental health problems; no medical care received for health problems; default on last month’s rent or mortgage; and class cancellations with no distance learning. Race/ethnicity, sex, income, and their intersections were used to measure distributional implications across historically marginalized populations; state-specific, time-varying population mobility was used to measure lockdown intensity. Logistic regression models with pooled repeated cross-sections were used to estimate risk of dichotomous outcomes by social group, adjusted for confounding variables.

**Results:**

The 1 088 314 respondents (561 570 [51.6%; 95% CI, 51.4%-51.9%] women) were aged 18 to 88 years (mean [SD], 51.55 [15.74] years), and 826 039 (62.8%; 95% CI, 62.5%-63.1%) were non-Hispanic White individuals; 86 958 (12.5%; 95% CI, 12.4%-12.7%), African American individuals; 86 062 (15.2%; 95% CI, 15.0%-15.4%), Hispanic individuals; and 50 227 (5.6%; 95% CI, 5.5%-5.7%), Asian individuals. On average, every 10% reduction in mobility was associated with higher odds of unemployment (odds ratio [OR], 1.3; 95% CI, 1.2-1.4), food insufficiency (OR, 1.1; 95% CI, 1.1-1.2), mental health problems (OR, 1.04; 95% CI, 1.0-1.1), and class cancellations (OR, 1.1; 95% CI, 1.1-1.2). Across most dimensions compared with White men with high income, African American individuals with low income experienced the highest risks (eg, food insufficiency, men: OR, 3.3; 95% CI, 2.8-3.7; mental health problems, women: OR, 1.9; 95% CI, 1.8-2.1; medical care inaccessibility, women: OR, 1.7; 95% CI, 1.6-1.9; unemployment, men: OR, 2.8; 95% CI, 2.5-3.2; rent/mortgage defaults, men: OR, 5.7; 95% CI, 4.7-7.1). Other high-risk groups were Hispanic individuals (eg, unemployment, Hispanic men with low income: OR, 2.9; 95% CI, 2.5-3.4) and women with low income across all races/ethnicities (eg, medical care inaccessibility, non-Hispanic White women: OR, 1.8; 95% CI, 1.7-2.0).

**Conclusions and Relevance:**

In this cross-sectional study, African American and Hispanic individuals, women, and households with low income had higher odds of experiencing adverse outcomes associated with the COVID-19 pandemic and stay-at-home orders. Blanket public health policies ignoring existing distributions of risk to well-being may be associated with increased race/ethnicity–based, sex-based, and income-based inequities.

## Key Points

**Question:**

Were state-specific mobility restrictions during the first wave of the COVID-19 pandemic in the United States associated with the well-being of individuals in groups that have historically been marginalized on the basis of race/ethnicity, sex, or income?

**Findings:**

In this cross-sectional study of 1 088 314 US adults, African American individuals with low income, Hispanic individuals, and women of all racial/ethnic groups had higher risks of experiencing unemployment, class cancellations, food insufficiency, and mental health problems during the first wave of the COVID-19 pandemic.

**Meaning:**

These findings suggest that public health policies that ignore existing distributions of risks to well-being may be intrinsically regressive if they fail to target necessary relief measures to individuals who have historically experienced the most marginalization.

## Teaser

This cross-sectional study identifies and quantifies the association of race/ethnicity, sex, and income as well as state-specific lockdown measures with 6 well-being dimensions in the United States.

## Introduction

Since the COVID-19 outbreak was declared a global pandemic, the threat of the virus has been evolving and unpredictable. However, its disproportionate effect on populations that have been historically marginalized has been persistent and unsurprising.^1^ COVID-19 has highlighted systemic inequity as a planetary issue, a preexisting condition of disadvantage underlying the social structure of society, such that COVID-19 cannot be thought of as merely a health crisis. Structural and institutional disparities, particularly racial/ethnic disparities, are central to understanding the implications and mitigation of the disease.^2,3^ Increasingly, research, aided by ample historical precedents, indicates that the most socially marginalized populations are bearing the heaviest burden.^4^

During the early months of COVID-19 in the United States, stay-at-home orders were implemented in most states. The goal of these orders was to reduce the potential for personal contact, which can contribute to the spread of COVID-19.^5^ Statewide mandates, while varying in strictness, were blanket strategies that restricted movement by requiring citizens to stay home except to conduct essential tasks or business. These policies have been associated with important reductions in cases and deaths,^6^ although the benefits have been unequally distributed across the population. Groups that have historically been marginalized have disproportionately borne the burden of COVID-19–related infection and mortality^3^ as well as the unintended consequences of policies designed to prevent this disease burden.^7,8,9^ Given the biomedical nature of the pandemic, health research on pandemic-related inequities has largely been illness oriented, focusing on the disease burden^1,2,10^; however, a more broadly based epistemology of health^11,12^ recognizes that health and well-being depend on multiple factors (eg, social, environmental, economic) and require consideration of the latter incidental burdens.^11^ While evidence of the consequences of COVID-19 policies continues to grow,^8,9,13,14^ the distribution of risks to well-being associated with human mobility restrictions has yet to be modeled for the United States.^9^ Emergency response options have complex trade-offs between short-term and long-term consequences, between potential harms and benefits,^15^ all of which interact with existing disparities in well-being. Quantifying the heterogeneity of risks across the population can aid decision-makers in predicting and balancing consequences prior to policy implementation for more equitable COVID-19 pandemic outcomes.

The objective of this study was to estimate the associations between state-specific mobility restrictions and well-being across historically excluded groups, including those groups at the intersections of race/ethnicity, sex, and income categorizations.^16^ We hypothesized that the distribution of risks to well-being would reproduce patterns of social marginalization. We hypothesized that when mobility restrictions were high, the risk of COVID-19 spreading may have been lower and the risk of socioeconomic adversity higher.

## Methods

### Data

Data are from phase 1 of the Interagency Federal Statistical Rapid Response Survey to Measure Effects of the Coronavirus (COVID-19) Pandemic on the United States Household Population: 2020 Household Pulse Survey (HPS). The HPS used the US Census Bureau’s Master Address File to contact households via email, text message, and/or telephone and produce representative estimates at the national, state, and metropolitan area levels. A total of 1 088 314 successful interviews were completed via internet questionnaires between April 23 (week 1) and July 21 (week 12), 2020. Sample size was determined such that a 2 percentage point difference in weekly HPS survey estimates would be detectable for an estimated 40% of the population with a 90% confidence interval at each level.^17^ A Census Bureau Notice and Consent Warning was displayed prior to participation, and accessing the survey indicated participants’ consent.^18^ The data are publicly available and approved under the Office of Management and Budget.^19^ Further technical details are available on the Census Bureau website.^18^ A second data source was the publicly available COVID-19 projections of the Institute of Health Metrics and Evaluation (IHME). IHME provides modeled state-level estimates of (1) weekly case and death rates (sourced from the Johns Hopkins Coronavirus Resource Center^20^) and (2) movement of US state populations based on anonymous cell phone data from several technology companies (ie, Facebook, Google, SafeGraph, and Descartes Labs).^21^ Finally, we obtained population estimates stratified by race from the American Community Survey Demographic and Housing 2015 to 2019 Estimates of the Census Bureau.^22^ The study used secondary deidentified data, making it exempt from institutional review board review. This study follows the Strengthening the Reporting of Observational Studies in Epidemiology (STROBE) reporting guideline.

### Outcomes

We examined the association of the COVID-19 pandemic with important indicators of well-being (eTable 1 in the Supplement). For several survey items, the questionnaire was explicit in asking participants to self-report causality by attributing outcomes to the pandemic (eg, “due to the coronavirus pandemic” or “since March 13, 2020”). Because of this unambiguity, we regarded these outcomes as pandemic-related. In total, we studied 6 composite well-being indicators, including pandemic-related unemployment, attributed to contracting COVID-19, being afraid of spreading COVID-19, being laid off because of COVID-19, or a business closing because of COVID-19; pandemic-related food insufficiency, defined as there sometimes or often not being enough food to eat in the last 7 days or food availability becoming worse after March 13, 2020; mental health problems, defined as respondents having scores of 12 or greater on indices of anxiety, worry, lack of pleasure, and hopelessness^14^; pandemic-related inaccessibility of medical care for health problems, defined as the respondent needing medical care for something other than COVID-19 in the past 4 weeks but not receiving it because of the pandemic; default on last month’s rent or mortgage; and pandemic-related class cancellations among households with school-aged children, defined as classes normally taught in person at school cancelled without any distance learning option (eTable 1 in the Supplement). If the outcome could not be explicitly attributed to the COVID-19 pandemic, which included mental health problems and default on rent or mortgage, it was restricted to the extremes of its distributions to capture the most severe conditions (eTable 2 in the Supplement).

### Exposures

This study focused on 4 key exposures: race/ethnicity, sex, income, and mobility restrictions. Race/ethnicity was measured as the 3 dummy variables of African American, Hispanic, and Asian, with the non-Hispanic White population serving as the referent. In the HPS, race/ethnicity classifications were options defined by the Census Bureau and originally included for weighting survey estimates.^18^ Sex was measured as a dummy variable for female respondents. Income was measured by 2 dummy variables. Low-income households were those earning less than $35 000 per year, and lower-middle income households were those earning between $35 000 and $75 000 per year. The referent consisted of the comparatively higher-income households earning more than $75 000 per year.^23^

Mobility restriction measured the reduced potential for personal contact; these restrictions were designed to help to control viral spread.^5^ During the study period, mobility was dependent on the imposition of state-specific government mandates, including educational facility and business closures, stay-at-home orders, public gathering restrictions, and travel restrictions; therefore, it operated as an ecological-level exposure.^24^ We exploited within-state weekly variation to estimate the associations of mobility restrictions with the outcomes. Mobility restriction was expressed as a percentage, with 0% representing no restriction and 100% representing complete restriction on mobility (eFigure 1 and eFigure 2 in the Supplement).

Other covariates included age, education, marital status, and household size. State-specific and survey week–specific COVID-19 cases and deaths per 1000 were used as ecological-level controls^24^ in all models.

### Statistical Analysis

We conducted our analyses using 2 sets of regression models to study (1) differences in outcomes with respect to race, sex, and income independently and (2) interactions of race, sex, and income. In the first set of logistic regression models, we determined the associations of race/ethnicity, sex, income (individual level), and mobility (state level) with each of the 6 binary outcomes (individual level). We modeled mobility as a continuous variable, assuming the outcomes responded linearly to changes in mobility (eTable 3 in the Supplement). In addition to the individual-level and household-level covariates, we included a discrete variable indicating survey week, which controlled for trends common to all states during the study period. We included 50 state dummy variables to control for all unobserved time-invariant confounders operating at the state level, such as the potential confounder of state-specific political ideology. Conditional on controlling for state dummy variables, mobility changes are likely quasi-exogenous (eMethods in the Supplement). To account for time-varying pandemic severity, we included weekly state-level COVID-19 case and death rates. It is important to control for pandemic severity, given that evidence suggests that states with higher COVID-19 case and mortality rates experienced larger economic damage early in the pandemic (eMethods in the Supplement).^25^ We applied HPS sampling weights to our coefficient estimates, rendering them representative of the population.^26^ SE estimates are robust and corrected for clustering (sandwich estimator) at the state level to account for intrastate correlations.^27^

Beyond singular group-level differences, our second set of models examined the interactions between race/ethnicity, sex, and income to probe the social patterning of the outcomes and identify high-risk groups.^16^ We created 3-way interaction dummy variables between the categories, assigning the referent to non-Hispanic White men with high income. Sample sizes for each intersectional category are in eTable 4 in the Supplement. In all other aspects, the models are identical to those in the first analysis set.

Regressions were run separately for each composite outcome in Stata version 15 (StataCorp). Statistical significance was set at *P* < .05, and tests were 2-tailed. Our models are theoretically motivated, and we report odds ratios (ORs) or probabilities with confidence intervals for all factors,^28^ thereby minimizing the problems associated with multiple comparisons. We circumscribed results in terms of association; however, due to plausible within-state quasi-exogeneity of mobility changes that approximates random assignment,^29^ there is likely a strong correspondence between mobility restrictions and the outcomes.^30^ In sensitivity analyses, we (1) relaxed our assumption of a linear mobility dose response; (2) explored sensitivity to potential autocorrelation at the individual and state levels; (3) and compared point estimates sensitivity to weekly vs monthly mobility restriction measurements (eMethods in the Supplement).

## Results

Mobility restrictions varied across states and declined between April and July 2020. Mobility restrictions tended to be highest in April and ranged between 15% to 60% across states in the first week of the survey (eFigure 1 and eFigure 2 in the Supplement). A mean (SD) of 90 692.8 (22 305.2) individuals responded weekly, at an average response rate of 7.7% for a total of 1 088 314 respondents during the 12 weeks.^19^ Respondents were aged between 18 and 88 years of age (mean [SD] age, 51.55 [15.74] years), and 826 039 (62.8%; 95% CI, 62.5%-63.1%) were non-Hispanic White individuals; 86 958 (12.5%; 95% CI, 12.4%-12.7%), African American individuals; 86 062 (15.2%; 95% CI, 15.0%-15.4%), Hispanic individuals; and 50 227 (5.6%; 95% CI, 5.5%-5.7%), Asian individuals (Table 1). More than half of the respondents were married (626 307 [54.1%; 95% CI, 53.8%-54.4%]); 561 570 (51.6%; 95% CI, 51.4%-51.9%) were women; and nearly one-third held a bachelor’s degree (314 574 [16.8%; 95% CI, 16.7%-17.0%]) or graduate degree (273 966 [13.5%; 95% CI, 13.4%-13.7%]). A quarter of the households had low income (188 469 [24.4%; 95% CI, 24.1%-24.6%]), and 272 473 (26.4%; 95% CI, 26.2%-26.7%) had lower-middle income.

**Table 1.** Socioeconomic Characteristics of 1 088 314 Participants in Sample

Characteristic	Participants, % (95% CI)
Age, y	
18-34.9	27.6 (27.4-27.9)
35-44.9	17.7 (17.5-17.9)
45-54.9	16.2 (16.0-16.4)
55-64.9	17.4 (17.2-17.6)
≥65	21.0 (20.8-21.3)
Women	51.6 (51.4-51.9)
Men	48.4 (48.1-48.6)
Race	
Non-Hispanic White	62.8 (62.5-63.1)
African American	12.5 (12.4-12.7)
Asian	5.6 (5.5-5.7)
Hispanic	15.2 (15.0-15.4)
Other	3.9 (3.8-4.0)
Education	
<High school	2.6 (2.5-2.8)
Some high school	5.9 (5.7-6.1)
High school graduate	30.6 (30.3-30.9)
Some college	21.3 (21.1-21.5)
Associate degree	9.2 (9.0-9.3)
Bachelor’s degree	16.8 (16.7-17.0)
Graduate degree	13.5 (13.4-13.7)
Marital status	
Now married	54.1 (53.8-54.4)
Widowed	4.2 (4.1-4.3)
Divorced	11.9 (11.8-12.1)
Separated	2.3 (2.2-2.4)
Never married	26.9 (26.6-27.1)
Household income in 2019, $	
<$25 000	14.0 (13.7-14.2)
$25 000-$34 999	10.4 (10.2-10.6)
$35 000-$49 999	11.0 (10.8-11.2)
$50 000-$74 999	15.4 (15.3-15.6)
$75 000-$99 999	11.3 (11.1-11.4)
$100 000-$149 999	12.5 (12.4-12.7)
$150 000-$199 999	5.5 (5.5-5.6)
≥$200 000	5.9 (5.8-6.0)
Household members, mean (95% CI), No.	
Adults	2.8 (2.8-2.8)
Children	0.7 (0.7-0.7)

Table 2 shows the proportion of the population experiencing each well-being outcome; eTable 5 in the Supplement shows the estimated population affected. Among individuals who were not retired and willing to work, 18.8% (95% CI, 18.5%-19.0%; representing approximately 31.1 million individuals) experienced unemployment. Across all individuals, 22.4% (95% CI, 22.2%-22.7%; representing approximately 71.4 million individuals) experienced food insufficiency, 18.6% (95% CI, 18.3%-18.8%; representing approximately 60.8 million individuals) reported experiencing mental health problems, and 32.5% (95% CI, 32.2%-32.8%; representing approximately 107.2 million individuals) experienced inaccessibility of needed medical care for non–COVID-19 health problems in the previous month. Among those households with rent or mortgage payments, 15.1% (95% CI, 14.8%-15.3%; representing approximately 37.0 million individuals) did not pay last month’s rent or mortgage. Among households with children enrolled in school, 41.9% (95% CI, 41.4%-42.5%; representing approximately 29.5 million households) had class cancellations with no replacement of online or distance learning. There was a clear income gradient across all outcomes, with respondents with low income experiencing all domains at higher rates than respondents with lower-middle or high income. African American individuals experienced the highest burden of food insufficiency, inaccessibility of medical care, and rent or mortgage defaults. African American and Hispanic individuals experienced unemployment and cancellation of classes at similarly high rates. The prevalence of mental health problems was similar across all racial/ethnic groups.

**Table 2.** Prevalence of Outcomes Across Race/Ethnicity, Sex, and Income

Group	% (95% CI)^a^					
	Pandemic-related class cancellations	Pandemic-related inaccessibility of medical care	Pandemic-related food insufficiency	Pandemic-related job losses	Mental health problems	Default on rent or mortgage
Respondents, No.	263 848	980 088	1 088 314	876 756	987 139	729 530
All	41.9 (41.4-42.5)	32.5 (32.2-32.8)	22.4 (22.2-22.7)	18.8 (18.5-19.0)	18.6 (18.3-18.8)	15.1 (14.8-15.3)
Race/ethnicity						
Non-Hispanic White	38.8 (38.1-39.4)	32.4 (32.1-32.7)	19.3 (19.0-19.5)	16.4 (16.1-16.6)	17.6 (17.4-17.9)	10.9 (10.6-11.1)
African American	48.4 (47.0-49.9)	34.3 (33.5-35.2)	30.4 (29.7-31.2)	22.6 (21.8-23.3)	20.2 (19.4-20.9)	27.2 (26.3-28.1)
Hispanic	46.5 (44.9-48.1)	31.3 (30.4-32.2)	27.9 (27.1-28.7)	23.1 (22.3-24.0)	20.6 (19.8-21.4)	20.2 (19.3-21.1)
Asian	36.7 (34.7-38.8)	27.1 (26.0-28.2)	20.9 (19.9-22.0)	21.0 (19.9-22.2)	16.2 (15.2-17.3)	14.2 (13.0-15.5)
Other^b^	43.4 (41.1-45.7)	41.2 (39.9-42.6)	28.6 (27.3-29.8)	19.4 (18.2-20.6)	24.9 (23.7-26.1)	19.3 (18.0-20.6)
Income in 2019, $						
<35 000	50.9 (49.7-52.2)	35.4 (34.8-36.0)	34.3 (33.7-34.9)	26.9 (26.3-27.5)	27.3 (26.7-27.9)	25.1 (24.5-25.8)
35 000-75 000	44.9 (43.7-46.0)	33.1 (32.6-33.6)	23.6 (23.1-24.0)	20.8 (20.3-21.3)	18.9 (18.4-19.3)	16 (15.5-16.4)
>75 000	34.9 (34.2-35.5)	30.3 (30.0-30.6)	16.0 (15.7-16.2)	13.6 (13.4-13.9)	13.0 (12.8-13.3)	8.0 (7.8-8.3)
Sex						
Men	43.4 (42.5-44.3)	29.3 (28.9-29.7)	21.4 (21.0-21.7)	19.1 (18.7-19.5)	16.4 (16.0-16.7)	14.2 (13.8-14.6)
Women	40.7 (40.1-41.4)	35.5 (35.2-35.9)	23.4 (23.1-23.7)	18.4 (18.1-18.7)	20.6 (20.3-20.9)	15.8 (15.5-16.2)

^a^ Percentages reported in cells are probability-weighted and representative of the United States population aged 18 to 80 years.^22^

^b^ The Household Pulse survey defines the other category as “2 or more races plus other races, non-Hispanic.”

**Figure 1.**
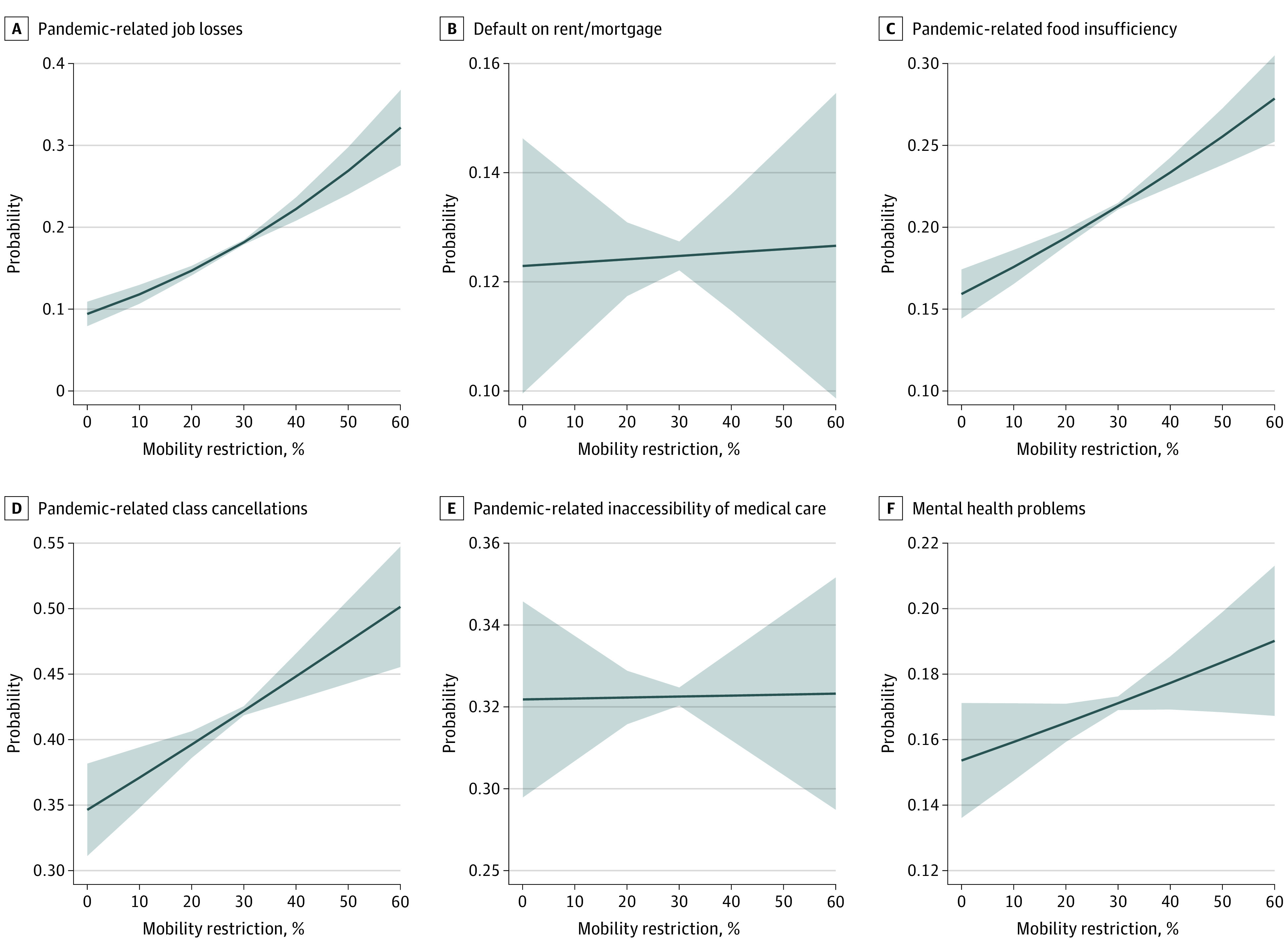
Associations Between Changes in Mobility Restriction and in Outcomes Solid lines represent probabilities from logistic regressions, holding all covariates at their sample means. Shaded areas represent 95% CIs. Mobility restriction represents within-state reductions in mobility based on week-to-week changes in mobility from normal levels. All models control for income, race/ethnicity, age, sex, education, marital status, numbers of individuals in the household, week of survey, state-level heterogeneity, and COVID-19 death and case rates. SE estimates are robust and clustered at the state level.

Mean (SD) mobility restriction was 24.8% (12.7) during the study period. In the fully adjusted logistic regression models with a linear mobility restriction exposure ranging from 1% to 60% (Figure 1), every 10% reduction in mobility was associated with higher odds of unemployment (OR, 1.3; 95% CI, 1.2-1.4), food insufficiency (OR, 1.1; 95% CI, 1.1-1.2), mental health problems (OR, 1.04; 95% CI, 1.0-1.1), and class cancellations (OR, 1.1; 95% CI, 1.1-1.2). Therefore, general population changes in these 4 outcomes were likely associated with the COVID-19 lockdown. Weekly mobility restrictions were not associated with inaccessibility of medical care or rent or mortgage defaults during the survey period. Our 3 sensitivity analyses yielded qualitatively similar findings. First, we found that the mobility dose responses for unemployment, class cancellations, and food insufficiency were linear (eFigure 3 in the Supplement). However, the association of mobility with mental health was attenuated. Second, the generalized estimating equation model, accounting for intra-individual correlations, provided similar estimates as our main model (eFigure 4 in the Supplement). Third, panel-data models, accounting for state-level autocorrelation, indicated that higher mobility restrictions were significantly associated with unemployment, food insufficiency, and class cancellations (eTable 6 in the Supplement). We found stronger correlations with mobility measured at the week level than at the month level.

**Figure 2.**
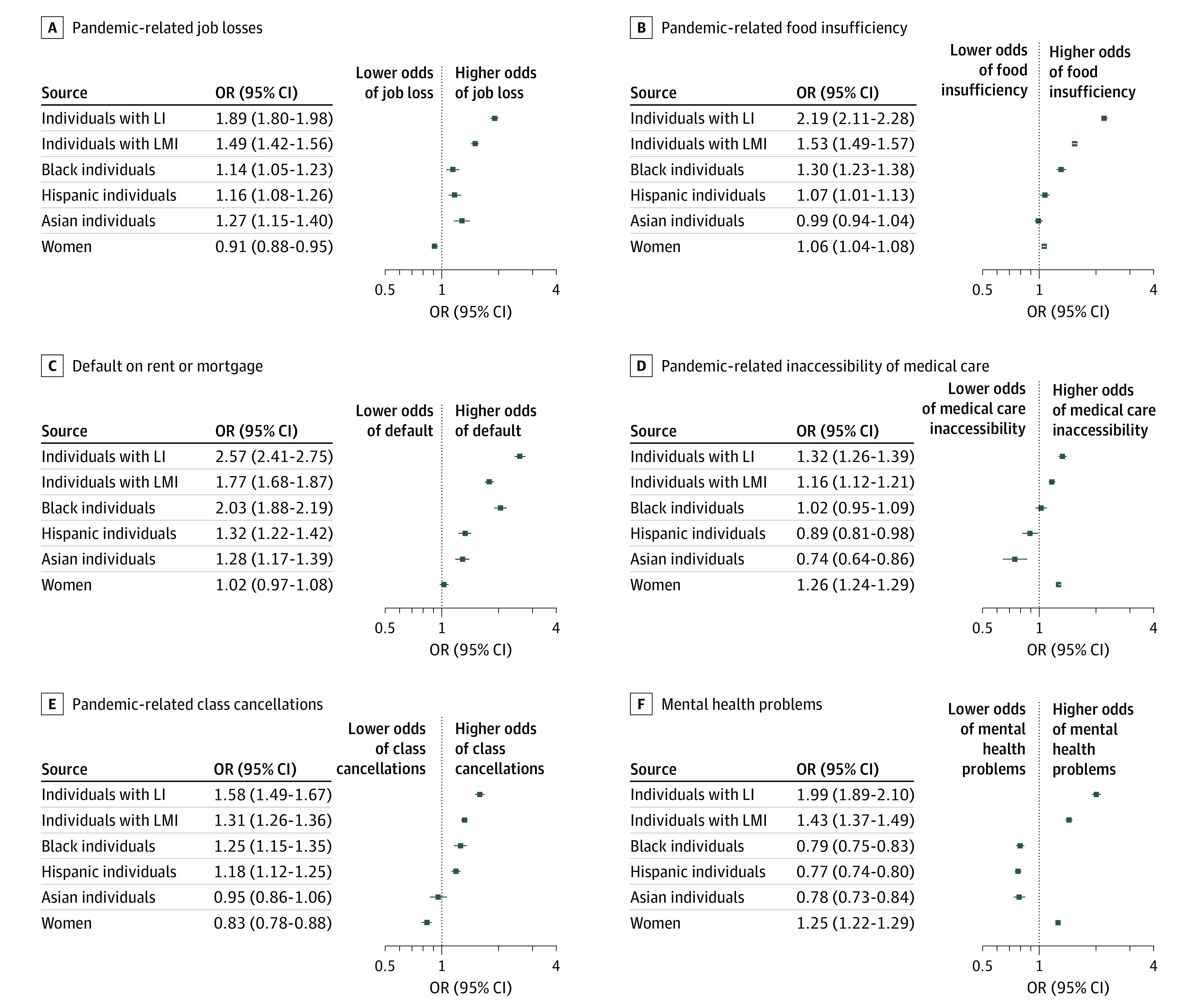
Risk of Outcome by Income, Race/Ethnicity, and Sex Dots are mutually adjusted point odds ratios, and whiskers represent 95% CIs. All models control for mobility, age, education, marital status, numbers of individuals in the household, week of survey, state-level heterogeneity, and COVID-19 death and case rates. SE estimates are robust and clustered at the state level. We provide more detail on the outcome descriptions in the legend for Figure 1. For African American, Hispanic, and Asian groups, the reference group was non-Hispanic White individuals. Low income (LI) was defined as households with income less than $35 000 per year; lower-middle income (LMI) are those with income between $35 000 and $75 000 per year. The reference group for low and lower-middle income groups is households with income greater than $75 000 per year.

Individuals with low income were at the highest risk of experiencing all outcomes: unemployment (OR, 1.9; 95% CI, 1.8-2.0), food insufficiency (OR, 2.2; 95% CI, 2.1-2.3), mental health problems (OR, 2.0; 95% CI, 1.9-2.1), inaccessibility of medical care (OR, 1.3; 95% CI, 1.3-1.4), rent or mortgage defaults (OR, 2.6; 95% CI, 2.4-2.8), and class cancellations (OR, 1.6; 95% CI, 1.5-1.7) (Figure 2). Controlling for income and other covariates, compared with non-Hispanic White individuals, African American individuals experienced considerably higher risks of food insufficiency (OR, 1.3; 95% CI, 1.2-1.4), rent or mortgage defaults (OR, 2.0; 95% CI, 1.9-2.2), and class cancellations (OR, 1.3; 95% CI, 1.2-1.4). Hispanic individuals and women also experienced higher risks than non-Hispanic White individuals and men, respectively.

**Figure 3.**
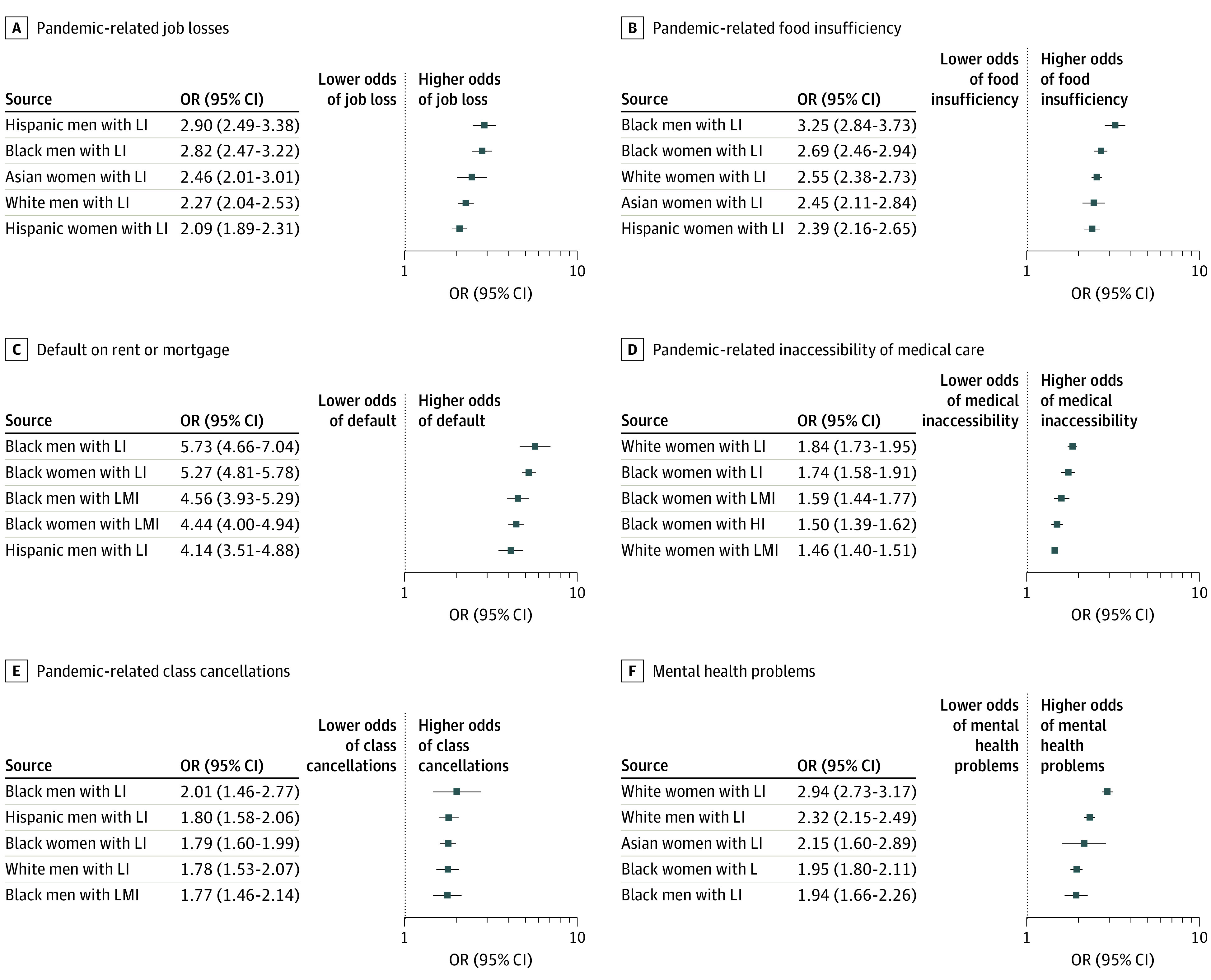
Intersectionality of Sex, Race/Ethnicity, and Income: Groups With Highest Risks of Outcomes Dots are mutually adjusted odds ratios and whiskers represent 95% CIs. Models included 3-way interaction dummy variables for sex, race, and income groups. The reference group was non-Hispanic White men with high income (HI; ie, >$75 000/year). All models control for mobility, age, education, marital status, numbers of individuals in the household, week of survey, state-level heterogeneity, and COVID-19 death and case rates. SE estimates are robust and clustered at the state level. We provide more detail on the outcome descriptions in the legend for Figure 1. Low income (LI) was defined as households with income less than $35 000 per year; lower-middle income (LMI) are those with income between $35 000 and $75 000 per year.

Across most dimensions, African American individuals with low income experienced the highest risks (food insufficiency, men: OR, 3.3; 95% CI, 2.8-3.7; women: OR, 2.7; 95% CI, 2.5-3.0; mental health problems, men: OR, 1.9; 95% CI, 1.7-2.3; women: OR, 1.9; 95% CI, 1.8-2.1; medical care inaccessibility, men: OR, 1.3; 95% CI, 1.1-1.5; women: OR, 1.7; 95% CI, 1.6-1.9; class cancellations, men: OR, 2.0; 95% CI, 1.5-2.8; women: OR, 1.8; 95% CI, 1.6-2.0; unemployment, men: OR, 2.8; 95% CI, 2.5-3.2; women: OR, 2.0; 95% CI, 1.8-2.3; and rent/mortgage defaults, men: OR, 5.7; 95% CI, 4.7-7.1; women: OR, 5.3; 95% CI, 4.8-5.7) (Figure 3). Non-Hispanic White women with low income experienced the highest comparative risk of inaccessibility of medical care (OR, 1.8; 95% CI, 1.7-2.0); notably, African American women with high income were also at a higher risk of this outcome (OR, 1.7; 95% CI, 1.6-1.9) than the reference group. Non-Hispanic White women with low income (OR, 3.0; 95% CI, 2.7-3.2) were the most likely to experience mental health problems compared with the reference group. Hispanic men with low income (OR, 2.9; 95% CI, 2.5-3.4) experienced a higher risk of unemployment than the reference group.

## Discussion

To our knowledge, this article provides the first population-representative distributional estimates of the associations of state-specific mobility restrictions with well-being. Our initial descriptions of population well-being suggested that low-income and African American populations were the most affected during the lockdowns across all outcomes. For several outcomes, while mobility restrictions significantly increased the risks for the average individual, wide confidence intervals indicated that these increases were highly heterogeneous across the population. Exploring this heterogeneity further, our first level of exposure stratification reaffirmed the findings from the initial well-being descriptions. Our second exposure stratification uncovered the intersecting identities most at risk of experiencing the outcomes, showing multiplicative differences in the ORs between those experiencing the most and least disadvantage across all outcomes. Women constituted 60% of these highest-risk groups; among these, African American women experienced the most risks to well-being.

In this study, we approached health from a well-being perspective and explored outcomes beyond the usual bounds of health and health care. We recognized conditions of unemployment, rent or mortgage defaults, and class cancellations as important,^31^ given that they can be upstream drivers of health.^32^ This paradigmatic move upstream enabled our analysis to better highlight racism at the headwaters of many health inequities.^33^ For example, unemployment is a distal factor associated with rates of overall mortality.^32,34,35^ Prior to the COVID-19 pandemic, and during the lockdowns (as evidenced in our results), unemployment rates among African American and Hispanic individuals exceeded overall national rates.^36^ Previous research has also shown that housing disparities are related to health, notably to the mental and physical health of African American individuals with mortgage strain,^37,38,39^ an unfortunately prevalent situation in our results, albeit with qualifications. Contributing to the literature showing a positive association between education and health,^40^ new studies have found an association between COVID-19–related education disruptions (including school nutrition^41^ and counselling service^42^ suspensions) to weight gain^41^ and mental health problems^42^ among adolescents, particularly among African American and Hispanic youth, groups that were also at higher risk in our results.

Our results also support existing evidence of sex as an intersecting component of health inequities.^43^ We found that women of all races/ethnicities with low income had some of the highest risks, with African American women at the highest risk of food insufficiency and inaccessibility of medical care. African American women with high income were also at a high risk of inaccessibility of medical care. Prior to the lockdowns, food insecurity^44,45^ and poor access to medical care,^46^ such as to prenatal care^47^ and cancer treatment,^48^ were prevalent among African American women. In addition, we found that non-Hispanic White individuals experienced the highest risk of mental health problems, which is consistent with prelockdown evidence of their likelihood of having^49^ and reporting^50^ mental health symptoms.

These findings support the notion that racism is a public health issue^51^ and suggest that blanket policies can be regressive if they ignore existing distributions of risks to well-being.^52^ Our results support the assertion of Ogedegbe and colleagues that “existing structural determinants—including inequality in housing, access to care, differential employment opportunities, and poverty—that remain pervasive”^10^ in African American and Hispanic communities align with the distribution of risks associated with COVID-19 policies. While mobility restrictions may be necessary to counter public health threats, underlying distributions of risk to well-being should inform corrective actions prior to implementation to minimize possible adverse distributional implications.

Even after the need for mobility restrictions has passed for the COVID-19 pandemic, recovery from the first stay-at-home orders may continue to be compounded by tenacious health disparities, which may require long-term corrective actions.^53,54^ Given persistent inequality in the United States,^55^ relief measures for those experiencing the greatest risks to well-being should not have arbitrary end dates. Instead, they should remain in place until economic indicators signal a robust recovery.^56^ Both during and after lockdowns, we encourage race/ethnicity–conscious, sex-conscious, and income-conscious policies for targeting assistance to promote health equity.^57^

### Strengths and Limitations

This study has many strengths. First, we combined data sets with unique advantages, including representativeness, temporality, and COVID-19–pandemic relevance. Second, we established a plausible case for attribution given explicit survey language that led to self-reported causality by survey respondents. Additionally, the use of rigorous panel data techniques with large-sample repeated cross-sections lent confidence to our estimates. Third, by using intersectionality to identify those groups with the most risk, this analysis can improve public health policy targeting. Fourth, this study is relevant to current policy discussions on COVID-19 responses and racism as a public health issue.

This study is not without limitations. First, despite explicit survey language, there are inherent concerns associated with participants’ subjective reports of attribution. In the face of negative outcomes, participants may feel motivated to search for causal explanations^58^ and may choose to attribute their current state to the COVID-19 pandemic. Second, 2 outcomes, ie, default on rent or mortgage and inaccessibility of medical care, were not associated with mobility restrictions. Therefore, their significant adverse implications cannot be attributed directly to first-wave lockdowns. For these outcomes, we are less confident in the magnitudes of the associations, given the absence of baseline data. Third, we were unable to explore the interaction of mobility restrictions with subgroups because mobility responses to lockdowns are differential across socioeconomic groups, and mobility data with such granularity were unavailable.^59^

## Conclusions

In this study, we found that groups that have historically been marginalized on the basis of race/ethnicity, sex, and income had higher risks of experiencing 6 negative well-being outcomes. Blanket public health policies that ignore existing distributions of risk to well-being may be associated with increased race/ethnicity–based, sex-based, and income-based inequities.

## Notes

Supplement. **eMethods.** Supplementary Methods **eFigure 1. **Trend in Mobility Restriction Averaged Across US States in 2020 **eFigure 2.** Trend in Mobility Restriction Disaggregated by US States by Week of 2020 Household Pulse Survey **eFigure 3. **Nonlinear Association Between Changes in Mobility Restriction and Changes in Outcomes **eFigure 4. **Association Between Changes in Mobility Restriction and Changes in Outcomes Using Generalized Estimating Equations to Account for Within-Person Correlation **eTable 1. **Definitions of Final Outcomes **eTable 2. **Distributions of Intermediate Outcomes **eTable 3. **Levels of Measurement in Outcomes and Covariates in the Merged Data Set **eTable 4. **Intersectional Category Sample Sizes **eTable 5. **Estimated Populations Affected, Calculated Using ACS Data **eTable 6. **Panel-Data Regressions Using State-by-Week Averages of HPS Outcomes and Mobility Restriction **eReferences.**
