# Supplementary material for: Association of Human Mobility Restrictions and Race/Ethnicity–Based, Sex-Based, and Income-Based Factors With Inequities in Well-being During the COVID-19 Pandemic in the United States
Source: JAMA Netw Open. 2021 Apr 7;4(4):e217373. doi: 10.1001/jamanetworkopen.2021.7373 (PMC8027913; doi:10.1001/jamanetworkopen.2021.7373)
Supplement: Supplement. — eMethods. Supplementary Methods eFigure 1. Trend in Mobility Restriction Averaged Across US States in 2020 eFigure 2. Trend in Mobility Restriction Disaggregated by US States by Week of 2020 Household Pulse Survey eFigure 3. Nonlinear Association Between Changes in Mobility Restriction and Changes in Outcomes eFigure 4. Association Between Changes in Mobility Restriction and Changes in Outcomes Using Generalized Estimating Equations to Account for Within-Person Correlation eTable 1. Definitions of Final Outcomes eTable 2. Distributions of Intermediate Outcomes eTable 3. Levels of Measurement in Outcomes and Covariates in the Merged Data Set eTable 4. Intersectional Category Sample Sizes eTable 5. Estimated Populations Affected, Calculated Using ACS Data eTable 6. Panel-Data Regressions Using State-by-Week Averages of HPS Outcomes and Mobility Restriction eReferences. [file jamanetwopen-e217373-s001.pdf]

Supplemental Online Content

Chakrabarti S, Hamlet LC, Kaminsky J, Subramanian SV. Association of human mobility restrictions and race/ethnicity–based, sex-based, and income-based factors with inequities in well-being during the COVID-19 pandemic in the United States. *JAMA Netw Open*. 2021;4(4):e217373. doi:10.1001/jamanetworkopen.2021.7373

eMethods. Supplementary Methods
eFigure 1. Trend in Mobility Restriction Averaged Across US States in 2020
eFigure 2. Trend in Mobility Restriction Disaggregated by US States by Week of 2020 Household Pulse Survey
eFigure 3. Nonlinear Association Between Changes in Mobility Restriction and Changes in Outcomes
eFigure 4. Association Between Changes in Mobility Restriction and Changes in Outcomes Using Generalized Estimating Equations to Account for Within-Person Correlation
eTable 1. Definitions of Final Outcomes
eTable 2. Distributions of Intermediate Outcomes
eTable 3. Levels of Measurement in Outcomes and Covariates in the Merged Data Set
eTable 4. Intersectional Category Sample Sizes
eTable 5. Estimated Populations Affected, Calculated Using ACS Data
eTable 6. Panel-Data Regressions Using State-by-Week Averages of HPS Outcomes and Mobility Restriction
eReferences.

This supplemental material has been provided by the authors to give readers additional information about their work.

© 2021 Chakrabarti S et al. *JAMA Network Open.*

eMethods. Supplementary Methods

Data merging

Pooling twelve waves of weekly HPS data, we construct a data set that is a repeated cross-section at the individual/household level and a representative panel at the week-state level. In the HPS, each household is interviewed up to three times. As we treat the data as pooled repeated cross-sections, we do not need to consider the issue of loss to follow-up. We only analyze complete cases, and given that our sample size is large, our estimates are unlikely to be influenced substantially by this decision. Please see the HPS technical documentation for more information on how the Census Bureau handles missing data.[1] We merge these pooled HPS data with the IHME data on mobility, case rates, and death rates by week of survey and state of residence. Thus, in our final data set, all outcomes and covariates from the HPS vary at the individual/household level, while the mobility, case rates, and death rates vary at the state level, operating as ecological exposures (eTable3). For example, all individuals sampled from California in the first week of the HPS are assigned the same level of mobility, and their mobility changes weekly (twelve times corresponding to the twelve weekly surveys of the HPS).

Statistical model

For individual/household h in state s in week of survey t, we estimate multivariable logistic regression models as:

$$\ln \frac{P(Y_{hst} = 1)}{1 - P(Y_{hst} = 1)} = \alpha_k + \beta_1 MobRes_{st} + \sum_{j=2}^{J} \beta_j X_{jhst} + \delta_t + \gamma_s + \pi_{st}$$

© 2021 Chakrabarti S et al. *JAMA Network Open.*

where $P(Y_{hst} = 1)$ denotes the probability that the (individual/household level) composite outcome Y_{hst} takes on a value 1 for individual/household h in state s in week t. $MobRes_{st}$ represents a percentage-point increase in mobility restriction and varies by state and week of survey. The mobility restrictions variable is quasi-exogenous in that it is exogenous conditional on controlling for state fixed effects (state dummies). Mobility restrictions would be purely exogenous if any changes in restrictions were due to forces external to the model (e.g., if mobility restrictions were randomly assigned); however, state-specific political ideology may be associated with both mobility restrictions and the outcomes of interest. As an exogenous mobility restrictions variable would improve the plausibility of causality, we account for the confounder of political ideology via state dummy variables. J covariates are included at the individual/household level (X_{jhst}). These covariates include the respondents' race/ethnicity, sex, income, education, marital status, and the numbers of individuals living in the household. δ_t is a continuous variable coded 1-12 representing weeks of the survey, that controls for linear temporal changes in outcomes and covariates that are common across all states. γ_s is a vector of state dummy variables that adjusts for all unobserved time-invariant confounders operating at the state level. π_{st} represents COVID-19 case and death rates to control for economic damage[2] from high incidence rates that would have negative associations with our outcomes, the omission of which would overestimate the negative association with mobility restriction. In other words, omitting incidence rates from the regression model might bias mobility coefficients away from the null, assuming outcomes may have trended negatively even without mobility restrictions.

© 2021 Chakrabarti S et al. *JAMA Network Open.*

Caveats for differing levels of measurement across outcomes

There are differences in the levels of measurement of the exposures and outcomes. For food insufficiency, class cancellations, and default on rent/mortgage (eTable3) in which the respondent answers on behalf of the household and him/herself, it is reasonable to assume these outcomes operate at the household and individual levels. For example, we interpret any differences in the class cancellations outcome across sex as being between the children of female versus male respondents. However, given that the question on unemployment asks about the respondent or anyone in the household, and the individual may not be the one who is unemployed, there may be measurement error in the sex gradient when regressing unemployment on sex. As we do not except differential misclassification by sex, any biases for sex differences in unemployment are likely to drive coefficients towards the null.

Sensitivity analyses

Relaxing the assumption of linearity for mobility dose response

In a sensitivity analysis, we relax the assumption of a linear relationship between mobility restriction and outcomes. We recategorize mobility into five dummy variables: 10-19%, 20-29%, 30-39%, 40-49%, and ≥50% restrictions, with 0-9% as the reference category. The range of categories is appropriate given mobility restrictions vary between 0 and 60% in our sample. We fit regression models with these dummy variables instead of continuous mobility. This specification allows us to flexibly gauge the dose-response relationships of interest across a range of mobility restrictions. The association of mobility restrictions with some outcomes may be attenuated in this semi-parametric

© 2021 Chakrabarti S et al. *JAMA Network Open.*

mobility specification, given that extreme values have higher leverage in the linear specification. However, we interpret these results with caution, given the loss of information by binning the mobility data, which reduces variation and weakens the ability to detect nuanced relationships.

Autocorrelation/non-independence

Given that the HPS allows individuals to be resampled up to three times during the twelve weeks, we consider the potential issue of autocorrelation, which occurs when observations from the same individual are correlated. While autocorrelation is typically a problem in data sets with long time periods (time points per individual > 50),[3] we check that correlation between repeat observations is not significantly biasing our mobility estimates. We use a Generalized Estimating Equations (GEE) approach that explicitly models intra-individual correlations and corrects for biases stemming from possible autocorrelation or non-independence.[4]

Beyond the individual level, another possible source of autocorrelation is due to the fact that our data are structured as repeated cross-sections at the state-level. To gauge the degree of this bias, we collapse our data set to the state-week level and run regressions with state-level mean estimates of the outcomes and covariates (N=612 for 51 states and twelve weeks). We run our main regression models in a panel-data setting with ordinary least squares, accounting for state fixed effects, autocorrelation, and heteroscedasticity.

Recall differences across outcomes

© 2021 Chakrabarti S et al. *JAMA Network Open.*

For the models with state-level averages, we also check sensitivity of results to outcome recall periods. We execute one set of regression models with mobility measured weekly, similar to our logistic models at the individual level. In a second set of models, we use monthly mobility, as some outcomes have longer recall periods than a week. We then compare whether our estimates at the state level are qualitatively similar to our main estimates at the individual/household level. A caveat to this check is that monthly mobility has less variation compared to weekly mobility and consequently may not correlate strongly with changes in outcomes over the weeks of the survey.

© 2021 Chakrabarti S et al. *JAMA Network Open.*

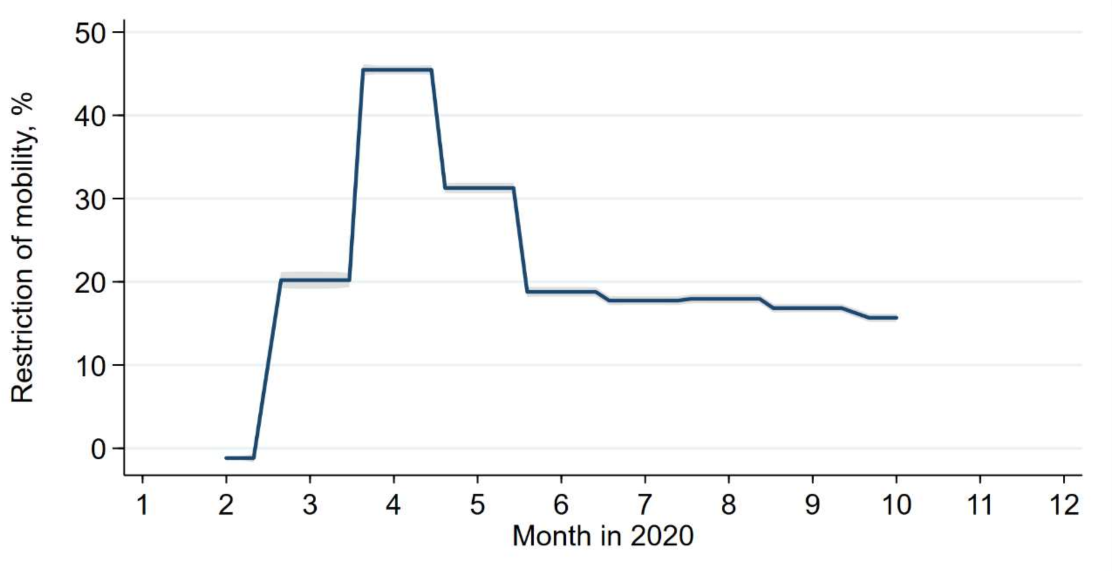

eFigure 1. Trend in Mobility Restriction Averaged Across US States in 2020
The mobility restriction variable is constructed from four data sources including Facebook, Google, SafeGraph, and Descartes Labs.[5] The indicator varies by week of 2020 Household Pulse Survey and US state. A higher value implies a higher restriction on mobility. It ranges between 0% (no restriction) and 100% (complete restriction on mobility).

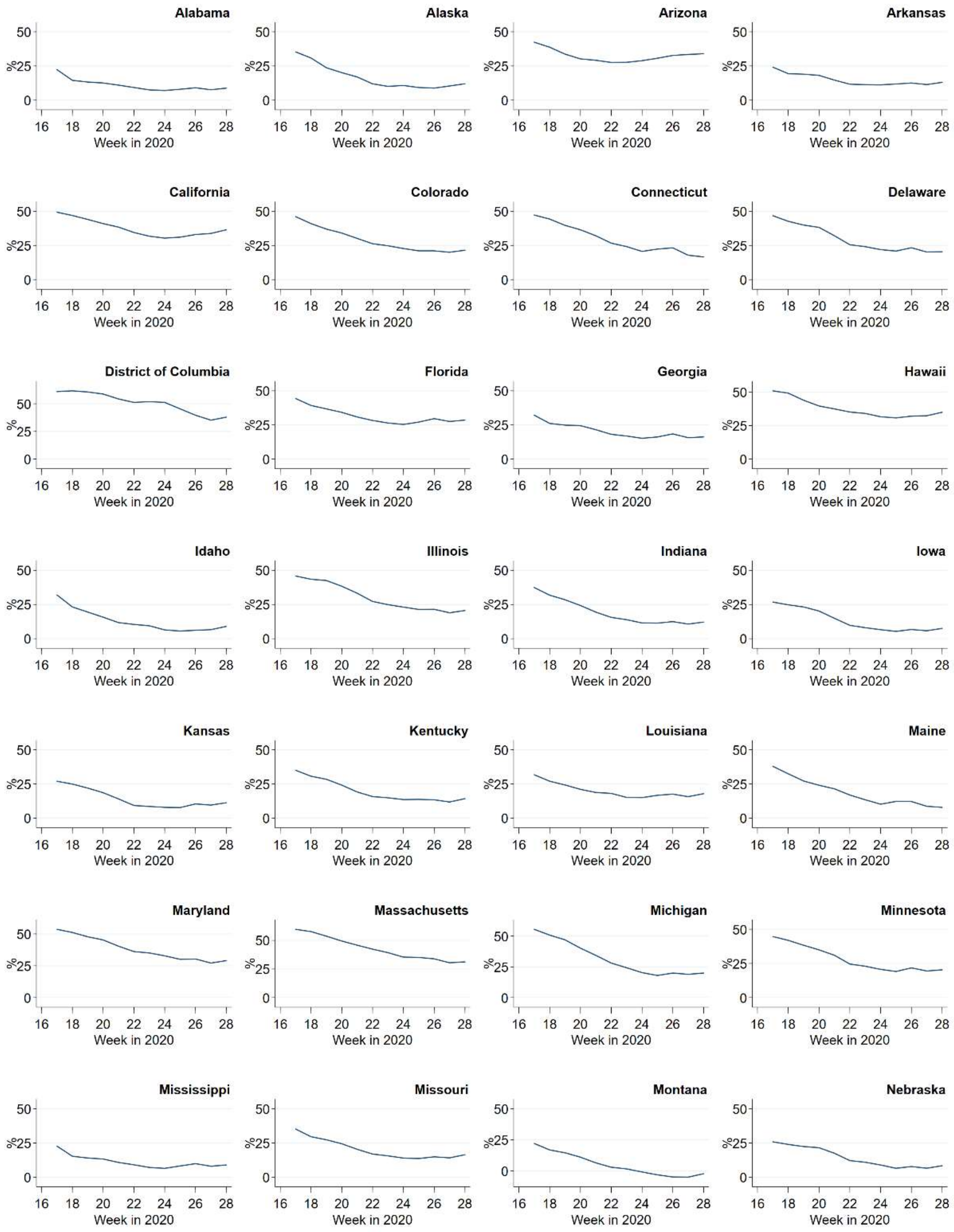

eFigure 2. Trend in Mobility Restriction Disaggregated by US States by Week of 2020 Household Pulse Survey

The mobility restriction variable is constructed from four data sources including Facebook, Google, SafeGraph, and Descartes Labs.[5] The indicator varies by week of 2020 Household Pulse Survey and US state. A higher value implies a higher restriction on mobility. It ranges between 0% (no restriction) and 100% (complete restriction on mobility). Week 16 refers to the week of April 22, 2020.

© 2021 Chakrabarti S et al. *JAMA Network Open.*

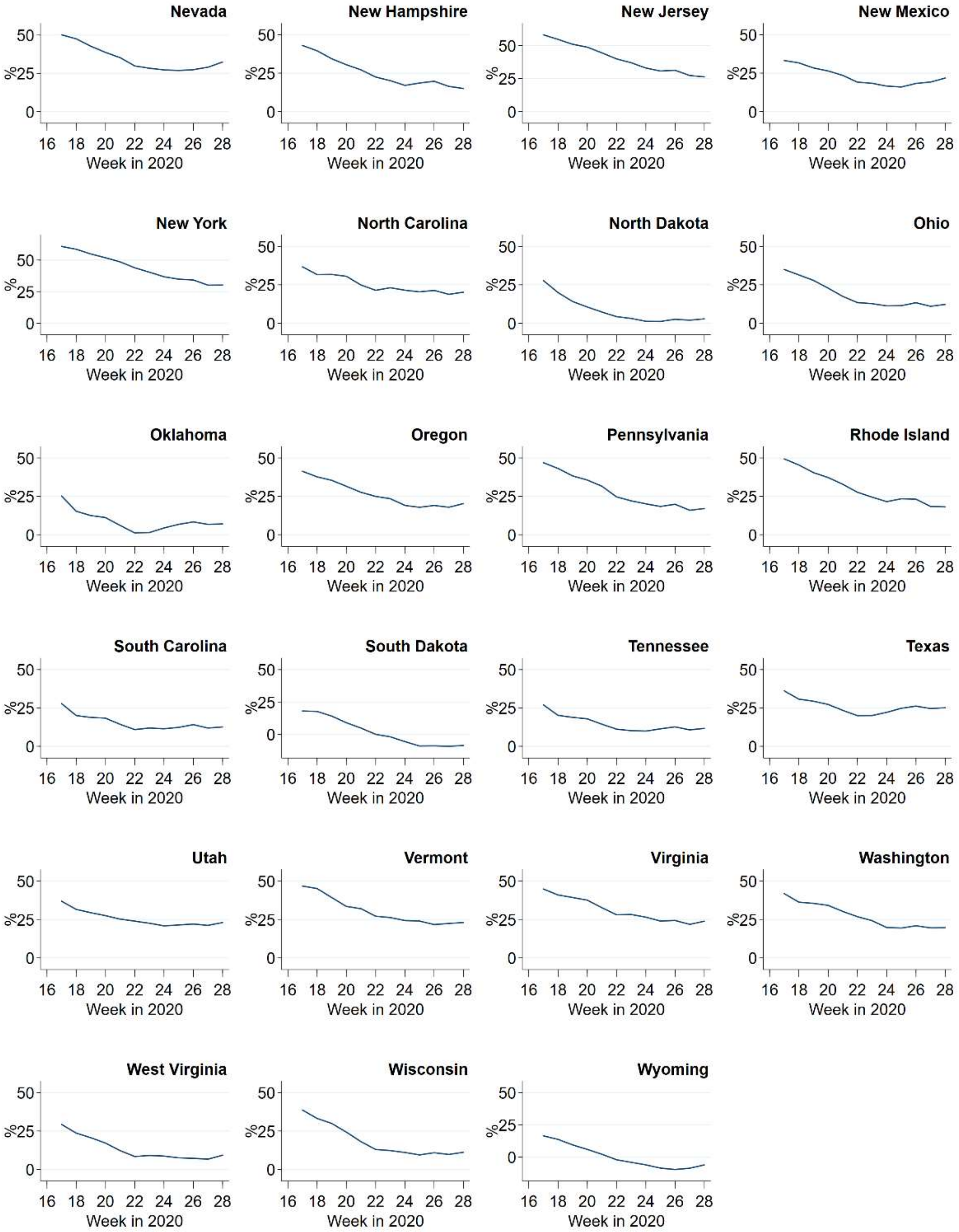

eFigure 2. Trend in mobility restriction disaggregated by US states by week of 2020 Household Pulse Survey (continued). The mobility restriction variable is constructed from four data sources including Facebook, Google, SafeGraph, and Descartes Labs.[5] The indicator varies by week of

© 2021 Chakrabarti S et al. *JAMA Network Open.*

2020 Household Pulse Survey and US state. A higher value implies a higher restriction on mobility. It ranges between 0% (no restriction) and 100% (complete restriction on mobility). Week 16 refers to the week of 22nd April 2020.

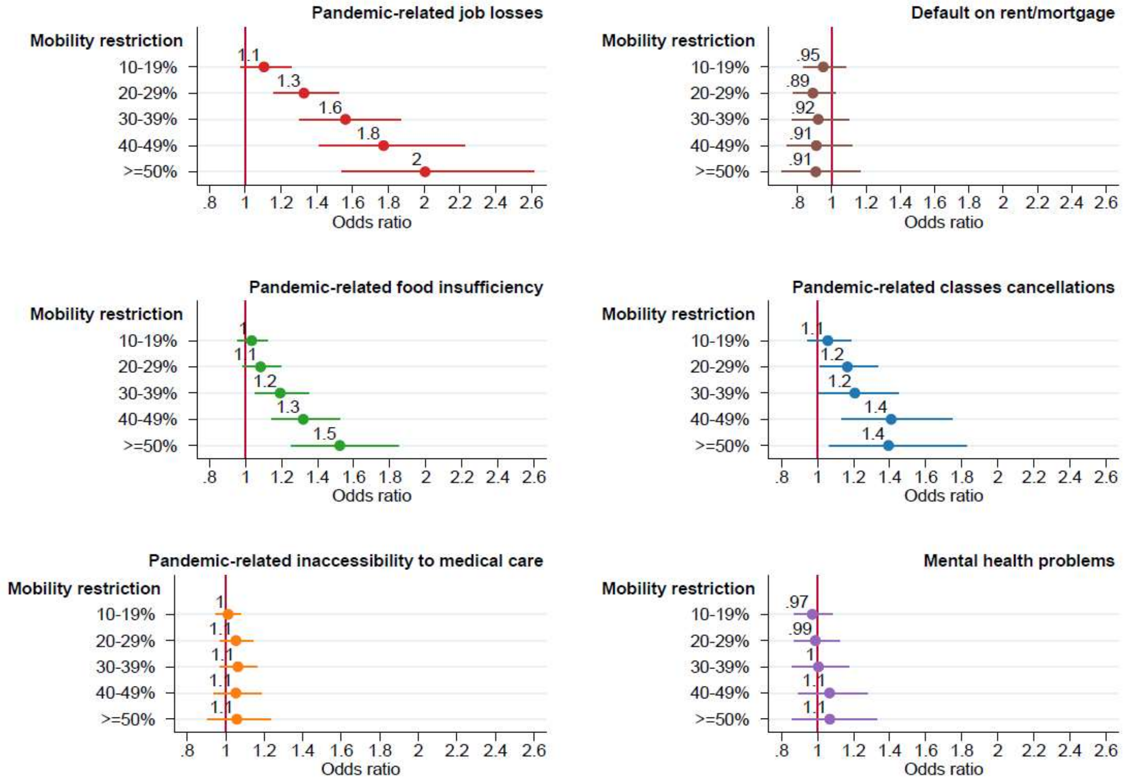

eFigure 3. Nonlinear Association Between Changes in Mobility Restriction and Changes in Outcomes

Dots represent point estimates. Whiskers represent 95% confidence intervals. Mobility restriction represents within-state reductions in mobility considering week-to-week changes in mobility from normal levels. All models control for income, race/ethnicity, age, sex, education, marital status, numbers of individuals in the household, week of survey, state-level heterogeneity, and COVID-19 death and case rates. Standard error estimates are robust.

© 2021 Chakrabarti S et al. *JAMA Network Open.*

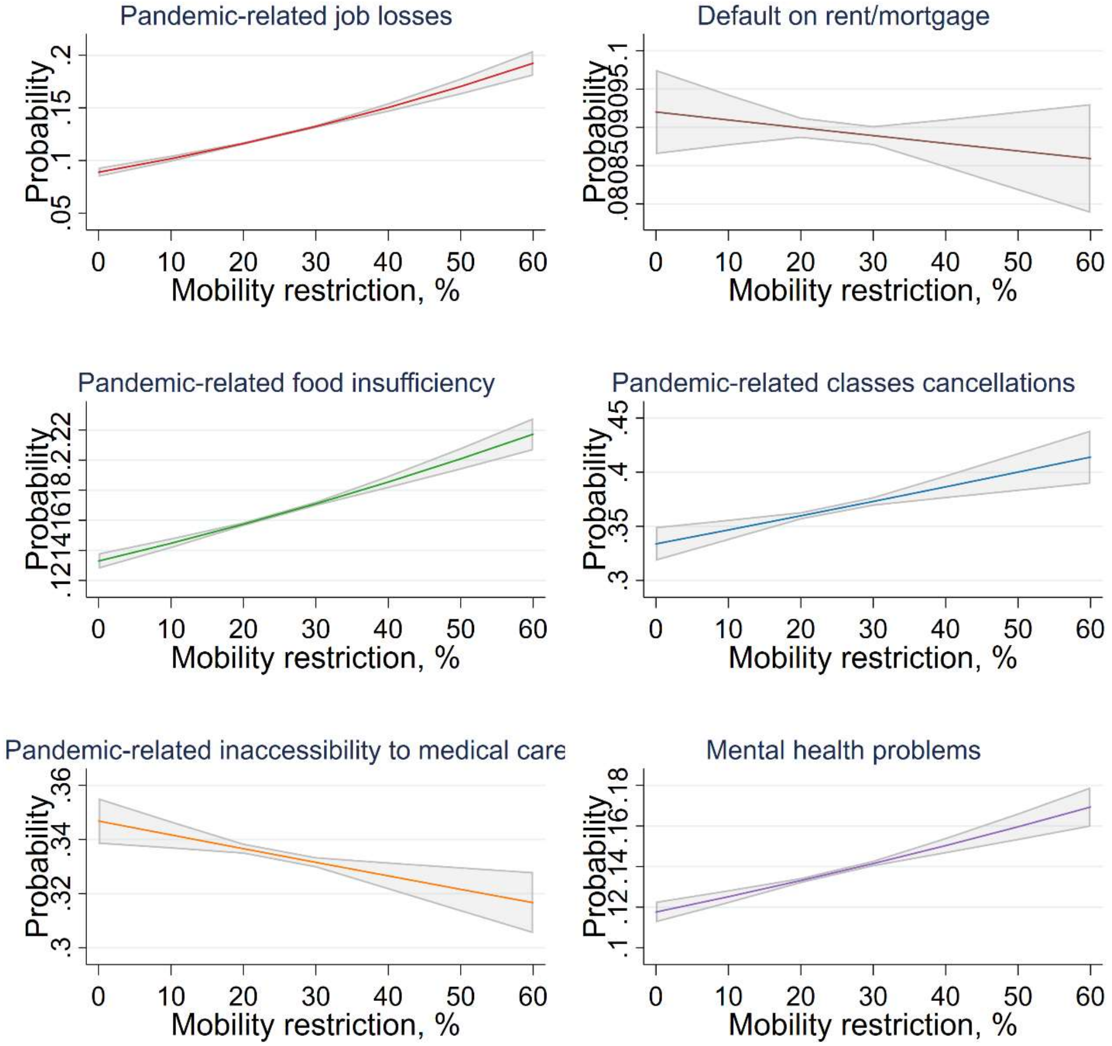

eFigure 4. Association Between Changes in Mobility Restriction and Changes in Outcomes Using Generalized Estimating Equations to Account for Within-Person Correlation

Solid lines represent predicted estimates. Grey shaded areas represent 95% confidence intervals. Mobility restriction represents within-state reductions in mobility considering week-to-week changes in mobility from normal levels. All models control for income, race/ethnicity, age, sex, education, marital status, numbers of individuals in the household, week of survey, state-level heterogeneity, and COVID-19 death and case rates. Estimates are corrected for correlation of observations among the same individual using a Generalized Estimating Equations model with an exchangeable correlation structure. Standard error estimates are robust.

© 2021 Chakrabarti S et al. *JAMA Network Open.*

eTable 1. Definitions of Final Outcomes

Outcome	Definition
**Self-reported causality**	
Unemployment (universe = population over the age of 18 years not retired and willing to work)	Respondent or anyone in household (not retired and willing to work) experienced a loss of employment since March 13, 2020 because of (1) sick with coronavirus symptoms **or** (2) caring for someone with coronavirus symptoms **or** (3) concerned about getting or spreading the coronavirus **or** (4) employer experienced a reduction in business (including furlough) due to coronavirus pandemic **or** (5) laid off due to coronavirus pandemic **or** (6) employment closed temporarily due to the coronavirus pandemic **or** (7) employment went out of business due to the coronavirus pandemic?
Food insufficiency (universe = all households)	In the last 7 days, household **sometimes or often** did not have enough to eat **or** household's **food security became worse after March 13**.
Classes cancelled (universe = households with school-aged children)	For children in the household, due to coronavirus pandemic classes normally taught in person at school were cancelled **without any replacement** with online/distance learning **or** other means.
No medical care (universe = all households with a valid response)	In the last 4 weeks, respondent needed medical care for something other than coronavirus but did not get it because of the pandemic.
**Non-self-reported causality**	
Default on rent or mortgage (universe = renters or household with a mortgage)	Household did not pay last month's rent or mortgage on time.
Mental health problems (universe = all households)	In the last 7 days, respondent **felt nervous/anxious** (score 1(never) - 4 (everyday)), **could not stop worrying** (score 1(never) - 4 (everyday)), **had little pleasure in doing** things (score 1(never) - 4 (everyday)), **felt depressed/hopeless** (score 1(never) - 4 (everyday)). Scores from the 4 dimensions were summed and the sum ranged from 4 (good mental health) to 16 (bad mental health)). Respondents with **scores ≥12** were considered to be those with mental health problems.

© 2021 Chakrabarti S et al. *JAMA Network Open.*

eTable 2. Distributions of Intermediate Outcomes

	N	Percent
**Main reason for not working for pay or profit**		
Did not want to be employed at this time	14,588	1.34
Was sick with coronavirus symptoms	4,159	0.38
Was caring for someone with coronavirus symptoms	1,074	0.10
Was caring for children not in school or daycare	24,349	2.24
Was caring for an elderly person	5,450	0.50
Was sick (not coronavirus related) or disabled	23,087	2.12
Retired	196,970	18.10
Employer experienced a reduction in business (including furlough) due to coronavirus pandemic	45,421	4.17
Was laid off due to coronavirus pandemic	31,784	2.92
Employment closed temporarily due to the coronavirus pandemic	37,571	3.45
Employment went out of business due to the coronavirus pandemic	4,921	0.45
Was concerned about getting or spreading the coronavirus	9,374	0.86
Other reason	60,715	5.58
No response	3,174	0.29
Employed in last 7 days	625,677	57.49
**Household food sufficiency for last 7 days**		
Enough of the kinds of food (I/we) wanted to eat	710,896	65.32
Enough, but not always the kinds of food (I/we) wanted to eat	288,326	26.49
Sometimes not enough to eat	54,817	5.04
Often not enough to eat	12,554	1.15
Question seen but category not selected	7,841	0.72
Missing / Did not report	13,880	1.28
**Household food sufficiency before March 13, 2020**		
Enough of the kinds of food (I/we) wanted to eat	827,299	76.00
Enough, but not always the kinds of food (I/we) wanted to eat	185,299	17.10
Sometimes not enough to eat	43,597	4.00
Often not enough to eat	10,603	1.00
Question seen but category not selected	13,880	1.30
Missing / Did not report	7,104	0.70
**Frequency of feeling nervous, anxious, or on edge over previous 7 days**		
Question seen but category not selected	5,407	0.50
Missing / Did not report	91,495	8.41
Not at all (score 1)	351,216	32.27
Several days (score 2)	352,881	32.42
More than half the days (score 3)	130,370	11.98
Nearly every day (score 4)	156,945	14.42
**Frequency of not being able to stop or control worrying over previous 7 days**		
Question seen but category not selected	5,957	0.55
Missing / Did not report	91,495	8.41
Not at all (score 1)	461,933	42.44
Several days (score 2)	314,188	28.87
More than half the days (score 3)	106,126	9.75
Nearly every day (score 4)	108,615	9.98
**Frequency of having little interest or pleasure in doing things over previous 7 days**		
Question seen but category not selected	6,480	0.60
Missing / Did not report	91,495	8.41

© 2021 Chakrabarti S et al. *JAMA Network Open.*

Not at all (score 1)	490,427	45.06
Several days (score 2)	310,543	28.53
	**N**	**Percent**
**Frequency of having little interest or pleasure in doing things over previous 7 days**		
More than half the days (score 3)	107,726	9.90
Nearly every day (score 4)	81,643	7.50
**Frequency of feeling down, depressed, or hopeless over previous 7 days**		
Question seen but category not selected	5,633	0.52
Missing / Did not report	91,495	8.41
Not at all (score 1)	504,899	46.39
Several days (score 2)	312,905	28.75
More than half the days (score 3)	89,491	8.22
Nearly every day (score 4)	83,891	7.71
**Delayed medical care not non COVID-19 illness due to pandemic in the last 4 weeks**		
Question seen but category not selected	4,441	0.41
Missing / Did not report	103,785	9.54
Yes	330,392	30.36
No	649,696	59.70
**Paid last month's mortgage or rent on time**		
Question seen but category not selected	1,341	0.12
Owned free and clear/occupied without payment of rent	357,443	32.84
Yes	649,502	59.68
No	57,209	5.26
Payment was deferred	22,819	2.10
**Pandemic impact on education: Classes normally taught in person at the school**		
No school-aged children in household	824,466	75.76
**Classes were cancelled**		
No	165,775	15.23
Yes	98,073	9.01
**Moved to a distance-learning format using online resources**		
No	53,848	4.95
Yes	210,000	19.30
**Moved to a distance-learning format using paper materials**		
No	208,298	19.14
Yes	55,550	5.10
**Classes changed in some other way**		
No	254,132	23.35
Yes	9,716	0.89
**School did not close**		
No	262,582	24.13
Yes	1,266	0.12

© 2021 Chakrabarti S et al. *JAMA Network Open.*

eTable 3. Levels of Measurement in Outcomes and Covariates in the Merged Data Set

			Levels of interpretation, as coded for analyses	
**Variable type**	**Data source**	**Coded in analyses**	**Unit of observation**	**Temporal scale for study period**
Outcome				
Unemployment	Household Pulse Survey (HPS)	Dummy variable	Anyone in household	Since March 13, 2020
Food insufficiency			Household	Last seven days
Classes cancelled			Children in household	Since February 2020
Inaccessibility to medical care			Respondent	Last four weeks
Default on rent/mortgage			Household	Last month's rent/mortgage
Mental health issues			Respondent	Last seven days
Exposure				
Race/ethnicity	HPS	Dummy	Respondent	Time-invariant
Sex		Dummy	Respondent	Time-invariant
Income		Dummy	Household	At time of survey
Mobility restrictions	Institute of Health Metrics and Evaluation (IHME), modelled using data from Facebook, Google, SafeGraph, and Descartes Labs	Continuous	State	Weekly since February 2020
Covariate				
Age	HPS	Continuous	Respondent	At time of survey
Education		Categorical	Respondent	At time of survey
Marital status		Categorical	Respondent	At time of survey
Household size		Continuous	Household	At time of survey
COVID-19 case rate	IHME modelled using source data from Johns Hopkins University (JHU)	Continuous	State	Weekly
COVID-19 death rate	IHME/JHU	Continuous	State	Weekly

eTable 4. Intersectional Category Sample Sizes

Intersectional category	Sample size
Male, Non-Hispanic White, high-income[a]	221,901
Female, Non-Hispanic White, high-income	275,353
Male, Non-Hispanic White, lower-middle-income[b]	78,394
Female, Non-Hispanic White, lower-middle-income	126,968
Male, Non-Hispanic White, low-income[c]	40,542
Female, Non-Hispanic White, low-income	82,881
Male, African American, high-income	13,010
Female, African American, high-income	23,663
Male, African American, lower-middle-income	7,111
Female, African American, lower-middle-income	16,460
Male, African American, low-income	6,119
Female, African American, low-income	20,595
Male, Hispanic, high-income	34,754
Female, Hispanic, high-income	23,737
Male, Hispanic, lower-middle-income	13,735
Female, Hispanic, lower-middle-income	14,074
Male, Hispanic, low-income	9,980
Female, Hispanic, low-income	15,028
Female, Asian, high-income	16,091
Female, Asian, lower-middle-income	5,341
Female, Asian, low-income	3,549
Male, Other, high-income	8,095
Female, Other, high-income	10,768
Male, Other, middle-income	3,823
Female, Other, lower-middle-income	6,567
Male, Other, low-income	3,011
Female, Other, low-income	6,764

[a]High-income households earn >$75,000 per year.
[b]Lower-middle-income households earn between $35,000 and $75,000 per year.
[c]Low-income households earn <$35,000 per year.

© 2021 Chakrabarti S et al. *JAMA Network Open.*

eTable 5. Estimated Populations Affected, Calculated Using ACS Data.[6]

	Unemployment (reference pop = current US labor force)[c]			Food insufficiency (reference pop = current US total pop)		
	**Eligible pop**	**Prop affected**	**Pop affected**	**Eligible pop**	**Prop affected**	**Pop affected**
**All**	163,555,585	0.19	31,075,561	324,697,795	0.22	71,433,515
**Female**	78,657,463	0.18	14,158,343	164,810,876	0.23	37,906,501
**Male**	76,307,461	0.19	14,498,418	159,886,919	0.21	33,576,253
**Low inc[a]**	43,545,144	0.27	11,757,189	91,240,080	0.34	31,021,627
**Low-mid inc[a]**	45,714,653	0.21	9,600,077	95,785,850	0.24	22,988,604
**White[b]**	112,336,093	0.16	17,973,775	235,377,662	0.19	44,721,756
**Black[b]**	19,679,601	0.23	4,526,308	41,234,642	0.3	12,370,393
**Hispanic**	27,909,802	0.23	6,419,254	58,479,370	0.28	16,374,224
**Asian**	8,554,489	0.21	1,796,443	17,924,209	0.21	3,764,084
	**Mental health problems (reference pop = current US total pop)**			**No medical care (reference pop = current US total pop)**		
	**Eligible pop**	**Prop affected**	**Pop affected**	**Eligible pop**	**Prop affected**	**Pop affected**
**All**	324,697,795	0.19	61,692,581	324,697,795	0.33	107,150,272
**Female**	164,810,876	0.21	34,610,284	164,810,876	0.36	59,331,915
**Male**	159,886,919	0.16	25,581,907	159,886,919	0.29	46,367,207
**Low inc[a]**	91,240,080	0.27	24,634,822	91,240,080	0.35	31,934,028
**Low-mid inc[a]**	95,785,850	0.19	18,199,311	95,785,850	0.33	31,609,330
**White[b]**	235,377,662	0.18	42,367,979	235,377,662	0.32	75,320,852
**Black[b]**	41,234,642	0.20	8,246,928	41,234,642	0.34	14,019,778
**Hispanic**	58,479,370	0.21	12,280,668	58,479,370	0.31	18,128,605
**Asian**	17,924,209	0.16	2,867,873	17,924,209	0.27	4,839,536
	**Default on rent/mortgage (reference pop = current US rent/mortgage)[d]**			**Classes cancelled (reference pop = students 3-19 years)[e]**		
	**Eligible pop**	**Prop affected**	**Pop affected**	**Eligible pop**	**Prop affected**	**Pop affected**
**All**	246,770,324	0.15	37,015,549	70,200,009	0.42	29,484,004
**Female**	118,677,254	0.16	18,988,361	33,760,722	0.41	13,841,896
**Male**	115,131,604	0.14	16,118,425	32,752,073	0.43	14,083,391
**Low inc[a]**	65,700,289	0.25	16,425,072	18,690,095	0.51	9,531,949
**Low-mid inc[a]**	68,973,613	0.16	11,035,778	19,621,274	0.45	8,829,574
**White[b]**	169,491,087	0.11	18,644,020	48,215,992	0.39	18,804,237
**Black[b]**	29,692,301	0.27	8,016,921	8,446,720	0.48	4,054,425
**Hispanic**	42,109,909	0.20	8,421,982	11,979,220	0.47	5,630,233
**Asian**	12,906,890	0.14	1,806,965	3,671,689	0.37	1,358,525

[a]Low-inc is the abbreviation for low-income households (< $35,000 per year), and low-mid inc is the abbreviation for low-middle-income households ($35,000-$75,000 per year). According the ACS Selected Economic Characteristics Table, 28.1% of households earn less than $35000 and 29.5% earn $35,000-$75,000 per year.
[b]White is the abbreviation for non-Hispanic White respondents, and Black is the abbreviation for African American

© 2021 Chakrabarti S et al. *JAMA Network Open.*

respondents. The population estimates by race are obtained from the ACS Demographic and Housing Estimates Table.
[c]Eligible population for unemployment is assumed to be the civilian labor force provided in ACS Selected Economic Characteristics Table.
[d]Per the Selected Housing Characteristics Table of the ACS there were 137,428,986 housing units; 36% of which were renter occupied. Of the remaining households, 62.7% have a mortgage. Thus, a total of 104,621,938 or 76% of households had an outstanding mortgage or rent.
[e]The ACS Demographic and Housing Estimates Table provides estimates for the age groups 0-4.9, 5-9.9, 10-14.9, and 15-19.9. We estimate the population of ages 3-19 as: (0-4.9)*0.4 + (5-9.9) + (10-14.9) + (15-19.9). We assume that the populations within one-year age intervals in the category 0-4.9 are approximately equally distributed.[7,8]

© 2021 Chakrabarti S et al. *JAMA Network Open.*

eTable 6. Panel-Data Regressions Using State-by-Week Averages of HPS Outcomes and Mobility Restriction

	**Pandemic-related job losses, %**	**Default on rent or mortgage, %**	**Pandemic-related food insufficiency, %**	**Pandemic-related classes cancellations, %**	**Pandemic-related inaccessibility to medical care, %**	**Mental health problems, %**
**Model 1**						
Weekly mobility change, %	0.31[a]	-0.017	0.11	0.23	0.013	0.12
	[0.22,0.39][b]	[-0.11,0.075]	[0.037,0.19]	[0.070,0.40]	[-0.065,0.091]	[0.032,0.21]
**Model 2**						
Monthly mobility change, %	0.25	-0.0090	0.076	0.13	-0.00	0.076
	[0.17,0.32]	[-0.084,0.067]	[0.0090,0.14]	[-0.031,0.29]	[-0.069,0.069]	[-0.010,0.16]
**N**	612	612	612	612	612	612
**States**	51[c]	51	51	51	51	51

[a]Coefficients interpreted as: percentage point change (+/-) in outcome per percentage point increase in mobility restriction.
[b]95% confidence intervals in brackets. All models control for income, race/ethnicity, age, sex, education, marital status, numbers of individuals in the household, week of survey, state fixed effects, and COVID-19 weekly death and case rates. Standard error estimates are heteroscedasticity- and autocorrelation-robust via clustering at the state level.
[c]We include the 50 states and the District of Columbia.

© 2021 Chakrabarti S et al. *JAMA Network Open.*

eReferences

1. JF Fields, Hunter-Childs J, Tersine A, et al. Design and Operation of the 2020 Household Pulse Survey, 2020. Published online 2020. https://www.census.gov/programs-surveys/household-pulse-survey/technical-documentation.html#phase1

2. Polyakova M, Kocks G, Udalova V, Finkelstein A. Initial economic damage from the COVID-19 pandemic in the United States is more widespread across ages and geographies than initial mortality impacts. *PNAS*. 2020;117(45):27934-27939. doi:10.1073/pnas.2014279117

3. Lebo MJ, Weber C. An Effective Approach to the Repeated Cross-Sectional Design. *American Journal of Political Science*. 2015;59(1):242-258. doi:https://doi.org/10.1111/ajps.12095

4. Wang M. Generalized Estimating Equations in Longitudinal Data Analysis: A Review and Recent Developments. Advances in Statistics. doi:https://doi.org/10.1155/2014/303728

5. IHME. COVID-19 resources. Institute for Health Metrics and Evaluation. Published March 24, 2020. Accessed October 13, 2020. http://www.healthdata.org/covid

6. ACS. American Community Survey. United States Census Bureau. Published 2019. Accessed February 25, 2021. https://www.census.gov/acs/www/data/data-tables-and-tools/american-factfinder/

7. The World Bank. Mortality rate, under-5 (per 1,000 live births) - United States | Data. Published 2019. Accessed February 27, 2021. https://data.worldbank.org/indicator/SH.DYN.MORT?locations=US

8. The World Bank. Fertility rate, total (births per woman) - United States | Data. Published 2018. Accessed February 27, 2021. https://data.worldbank.org/indicator/SP.DYN.TFRT.IN?locations=US

9. US Census Bureau. Source of the Data and Accuracy of the Estimates for the 2020 Household Pulse Survey. Published online 2020.

10. Statista Research Department. U.S. income distribution 2019. Statista. Published 2020. Accessed February 22, 2021. https://www.statista.com/statistics/203183/percentage-distribution-of-household-income-in-the-us/

© 2021 Chakrabarti S et al. *JAMA Network Open.*
